# Detection of HPV16 in Esophageal Cancer in a High-Incidence Region of Malawi

**DOI:** 10.3390/ijms19020557

**Published:** 2018-02-12

**Authors:** Anja Lidwina Geßner, Angelika Borkowetz, Michael Baier, Angela Göhlert, Torsten J. Wilhelm, Alexander Thumbs, Eric Borgstein, Lars Jansen, Katrin Beer, Henning Mothes, Matthias Dürst

**Affiliations:** 1 Department of General, Visceral and Vascular Surgery, Jena University Hospital—Friedrich-Schiller-University; 07747 Jena, Germany; anja.l.gessner@gmail.com; 2 Department of Gynecology, Jena University Hospital—Friedrich-Schiller-University, 07747 Jena, Germany; Lars.Jansen@med.uni-jena.de (L.J.); Katrin.Beer@med.uni-jena.de (K.B.); 3 Department of Urology, Technische Universität Dresden; 01307 Dresden, Germany; angelika@borkowetz.de; 4 Institute for Medical Microbiology, Jena University Hospital—Friedrich-Schiller-University, 07747 Jena, Germany; Michael.Baier@med.uni-jena.de; 5 Institute for Pathology, Jena University Hospital—Friedrich-Schiller-University, 07743 Jena, Germany; pathologiejena@gmail.com; 6 Department of Surgery, University Medical Centre Mannheim, 68167 Mannheim, Germany; tjwilhelm@web.de; 7 Department of Surgery, Queen Elizabeth Central Hospital—College of Medicine, Blantyre 3, Malawi; alex_thumbs@hotmail.com (A.T.); eborg@me.com (E.B.)

**Keywords:** human Papillomavirus, esophageal squamous cell carcinoma, p16^INK4a^ immunohistochemistry, Ki-67 proliferation index, polymerase chain reaction, in situ hybridization, alcohol, smoking

## Abstract

This study was designed to explore the role of human papillomavirus (HPV) in esophageal squamous cell carcinoma (ESCC). Fifty-five patients receiving diagnostic upper gastrointestinal endoscopy at Zomba Central Hospital or Queen Elizabeth Hospital in Blantyre (Malawi) in 2010, were included in our study. Formalin-fixed paraffin-embedded biopsies were collected for histopathological diagnosis. HPV DNA was detected using multiplex Quantitative PCR (qPCR) and in situ hybridization (ISH). p16^INK4a^ staining served as a surrogate marker for HPV oncogene activity. Cell proliferation was determined by Ki-67 staining. Human immunodeficiency virus (HIV) status was evaluated by serology. Data on the consumption of alcohol and tobacco, and history of tuberculosis (TBC), oral thrush, and Herpes zoster, were obtained by questionnaire. Forty patients displayed ESCC, three displayed dysplastic epithelium, and 12 displayed normal epithelium. HPV16 was detected in six ESCC specimens and in one dysplastic lesion. Among HPV-positive patients, viral load varied from 0.001 to 2.5 copies per tumor cell. HPV DNA presence could not be confirmed by ISH. p16^INK4a^ positivity correlated with the presence of HPV DNA (*p* = 0.03). Of particular note is that the Ki-67 proliferation index, in areas with diffuse nuclear or cytoplasmatic p16^INK4a^ staining ≥50%, was significantly higher in HPV-positive tumors compared to the corresponding p16^INK4a^ stained areas of HPV-negative tumors (*p* = 0.004). HPV infection in ESCC was not associated with the consumption of tobacco or alcohol, but there were significantly more patients drinking locally brewed alcohol among HPV-positive tumor patients compared to non-tumor patients (*p* = 0.02) and compared to HPV-negative tumor patients (*p* = 0.047). There was no association between HIV infection, history of TBC, Herpes zoster, oral thrush, or HPV infection, in ESCC patients. Our indirect evidence for viral oncogene activity is restricted to single tumor cell areas, indicative of the role of HPV16 in the development of ESCC. The inhomogeneous presence of the virus within the tumor is reminiscent of the “hit and run” mechanism discussed for β-HPV types, such as HPV38.

## 1. Introduction

Esophageal cancer (EC) is the eighth most common cancer diagnosis, with an incidence of 456,000 cases worldwide in 2012. More prevalent than other tumors, it shows high geographical variation, with high occurrence in a variety of regions, including the Asian esophageal cancer belt in Southcentral Asia, Turkmenistan, and parts of Southern Africa. Malawi belongs to the high incidence area of EC in Africa [1,2]. Although an epidemiological shift is taking place, with rising numbers of histologically proven adenocarcinoma of the esophagus in Western countries, the esophageal squamous cell carcinoma (ESCC) is still the predominant histopathological type worldwide [2]. This indicates that the incidence of EC is influenced by lifestyle and other exogenous factors. The impact of non-infectious agents, such as tobacco and alcohol, as well as infectious causes such as human papillomavirus (HPV), on the pathogenesis of ESCC is discussed in the literature [3,4].

In Malawi, ESCC is endemic, with an incidence rate of 24.2 per 100,000 residents, compared to 3.2 per 100,000 in the United States or 6.6 per 100,000 in the U.K. [1]. HPV prevalence in Eastern Africa is one of the highest in the world and exceeds 30% among women, even with normal cervical cytology [5]. HPV-related cervical cancer is the most frequent cancer type in Malawi with an incidence rate of 75.9 per 100,000 women [1].

Since the discovery of HPV type 16 in 1984, the role of HPV in human malignancies has become well established [6,7]. HPV is responsible for 2% of the cancer burden in developed regions and 7% of the cancer burden in developing regions [5]. High-risk HPV (hr-HPV) is known to play a causal role in the development of cervical, anal, head and neck cancer, and squamous cell carcinoma of the vulva [8,9]. HPV16 is the most prevalent representative in human cancers [10]. HPV is known for its strict tropism and commonly infects basal cells of stratified squamous epithelia [11]. HPV infection of the cervical epithelium is either productive or transforming in nature. Productive infections are characterized by koilocytes, typically seen in cervical intraepithelial neoplasia (CIN) 1 lesions. In productive infections, replication and expression of viral proteins are tightly regulated and are linked to the process of epithelial differentiation. Transforming viral infections are the result of virus persistence and the constitutive activity of viral oncogenes E6 and E7, which promote genetic instability. Accumulating genetic changes are a prerequisite for immortalization and tumorigenicity. Transforming infections usually result in CIN2/3 lesions, characterized by deregulated viral E6/E7 expression in the basal and parabasal layers, which distinguishes them from virus productive lesions [12]. Since the first report of HPV in esophageal tumors in 1982 [13], many studies have been conducted exploring the role of HPV in EC, but results remained inconclusive [14,15]. 

Tests for the detection of persistent HPV infections in tissue samples are manifold, and include in situ hybridization and target nucleic acid amplification. In particular, polymerase chain reaction (PCR) is used for HPV detection, genotyping, and viral load determination. In situ hybridization detects the viral genome within the histopathological context of a lesion [16]. Determination of the proliferation index, oncogene activity, and serological response provides further information on the role of HPV in ESCC. Ki-67 is a proliferation marker detected by immunohistochemistry and is commonly used to determine cell proliferation within tumor areas [17,18]. Immunohistochemical staining of p16^INK4a^ reflects the oncogene activity of the deregulated E7 oncoprotein, which inhibits retinoblastoma (Rb) [18,19]. 

To further explore the role of HPV in ESCC we applied different methods to investigate HPV infection in the tissue samples of patients with ESCC. Our study is the first to combine real-time qPCR, in situ hybridization, and the immunohistochemical staining of Ki-67 and p16^INK4a^ to determine HPV load, HPV prevalence within the histopathological context, the proliferation index, and oncogene activity, respectively. Moreover, further risk factors such as smoking, alcohol consumption, and human immunodeficiency virus (HIV) infection, were also considered. 

## 2. Results

A total of 55 patients attending upper gastrointestinal endoscopy in 2010 were included in this study. The tumor group consisted of 40 histologically proven ESCC patients. Three patients showed a dysplastic epithelium in their histopathology. For 12 patients, no histopathological signs of ESCC were found in their biopsies, therefore these patients were defined as non-tumor patients. 

### 2.1. Multiplex Real-Time PCR Assay

All 55 samples were examined using a multiplex real-time PCR assay for the detection of HPV16, 18, 31, and 45, in two independent runs. Since we could only detect HPV16 DNA, three further independent duplex real-time PCRs for the detection of HPV16 and β-globin were performed for most of the samples. For five samples, HPV16-positivity was reproducible in all runs, and for two samples it was reproducible in 4 of 5 and 3 of 5 runs, respectively. Thus, HPV16 DNA was detected in seven (12.7%) samples at least three times. These seven samples were considered HPV16-positive (Table 1). One of the 7 HPV-positive samples showed dysplastic epithelium. Six of these 7 HPV-positive biopsies stemmed from tumor patients. Therefore, HPV-positivity among patients with histopathologically confirmed ESCC was 15%. Of 15 samples, sufficient material was left for renewed DNA extraction and further HPV genotyping. For these samples, multiplex real-time PCR was performed for all 7 hrHPV types (HPV16, 18, 31, 33, 45, 52, and 58). HPV16-positivity could be confirmed in 2 of 3 cases. No other HPV genotypes could be detected. Reproducibility of the PCR results for HPV16 showed high overall agreement (Fleiss’s kappa = 0.6868, SE = 0.0348). Viral load, estimated by ∆C_T_ in a multiplex real-time PCR assay, in HPV-positive samples, varied from 0.001 to 1.01 HPV copies per cell. When normalizing for the proportion of tumor cells within the sections, viral load varied from 0.001 to 2.5 HPV copies per tumor cell. In four samples, viral load ranged from 1.3 to 2.5 HPV copies per tumor cell.

### 2.2. Histopathological Analysis, p16^INK4a^ Status, and the Ki-67 Proliferation Index

Six of the 7 HPV16-positive cases were tumor patients with histopathologically confirmed ESCCs. One HPV16-positive case showed dysplastic epithelium. The difference in HPV prevalence between tumor patients and non-tumor patients was not statistically significant (*p* = 0.3). Information about grading was available for 39 tumor patients. Grading of ESCC samples among HPV-positive tumor samples was significantly lower compared to HPV-negative tumor samples (*p* < 0.001). 

Evaluation of p16^INK4a^ and Ki-67 status was not possible for two HPV-negative and one HPV-positive sample, as there were no tumor cell areas visible in the stained section. 

**Figure 1:**
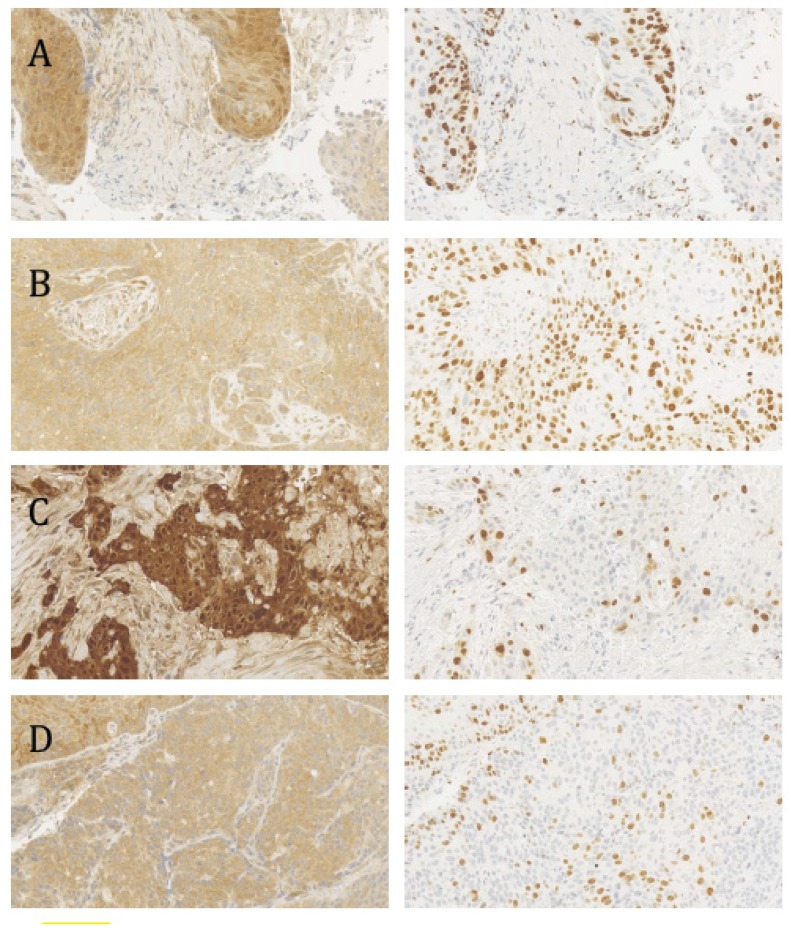
p16^INK4a^ and Ki-67 staining (left and right panels, respectively) in consecutive sections of human papillomavirus (HPV)-positive and HPV-negative tumor biopsies. (**A**) HPV-positive, diffuse nuclear p16^INK4a^ staining; (**B**) HPV-positive, diffuse cytoplasmatic p16^INK4a^ staining; (**C**) HPV-negative, diffuse nuclear p16^INK4a^ staining. (**D**) HPV-negative, diffuse cytoplasmatic p16^INK4a^ staining. The proliferation index (Ki-67 staining) is significantly higher in HPV-positive tumors. (**A**–**D**) were obtained with 20× magnification.

Three tumor samples showed a diffuse nuclear p16^INK4a^ staining pattern (overall ≥20% of tumor cell areas), of which one sample was HPV-positive (Figure 1, Table 1). Diffuse cytoplasmatic p16^INK4a^ positive staining (overall ≥20% of tumor cell areas) was found in 25 samples, of which 22 were tumor samples.

Biopsies showing diffuse nuclear or diffuse cytoplasmic p16^INK4a^ staining ˂20%, as well as biopsies with only focally stained cells, were defined as p16^INK4a^-negative. All HPV-positive tumor samples showed either diffuse nuclear or diffuse cytoplasmatic p16^INK4a^ staining ≥20%. Positive p16^INK4a^ staining was significantly associated with HPV status (*p* = 0.03). This association was still significant when positive nuclear or cytoplasmatic p16^INK4a^ staining ≥50% was taken as the threshold level for p16^INK4a^-positivity (*p* = 0.03). 

All HPV positive samples, overall, showed a high Ki-67 proliferation index. HPV-positive samples (median: 69.0%, interquartile range: 17.5%) had a higher proliferation index compared to HPV-negative tumors, including dysplastic samples (median: 52.0%, interquartile range: 25.8%; *p* = 0.03). Additionally, we determined the Ki-67 proliferation index exclusively in tumor areas with diffuse nuclear or cytoplasmatic p16^INK4a^ staining (Figure 1, Table 1). Interestingly, the Ki-67 proliferation index of HPV-positive samples (median: 69.5%, interquartile range: 27.0%) with nuclear or cytoplasmatic p16^INK4a^ staining ≥50% was significantly higher compared to the corresponding areas of HPV-negative tumor samples (median: 47.0%, interquartile range: 26.0%; *p* = 0.004). This association was also significant when the Ki-67 proliferation index of HPV-positive samples (median: 71.0%, interquartile range: 16.0%) with nuclear or cytoplasmatic p16^INK4a^ staining ≥20% was compared to HPV-negative tumor samples (median: 47.5%, interquartile range: 28.0%; *p* = 0.003). 

### 2.3. In Situ Hybridization

In situ hybridization was performed for all HPV-positive biopsies, except for one tumor, which lacked tumor cell areas in subsequent sectioning performed after histopathological diagnosis and HPV-genotyping. Moreover, HPV-negative tumors showing diffuse nuclear and cytoplasmic p16^INK4A^ staining, as well as tumors with only focal p16^INK4a^ staining, were also hybridized (Table 1). Only unspecific background in situ hybridization signals were seen, and no difference in signal patterns between HPV-positive and -negative tumor samples was observed. HPV16-positive cervical carcinomas and precancers were used as positive controls and showed the expected HPV DNA signal pattern (Figure S1, supplementary materials). 

### 2.4. Association between HPV Status, Clinical Data and Risk Factors

Clinical data regarding history of TBC, oral thrush, and Herpes zoster, as well as tobacco and alcohol consumption, were collected. These results are summarized in Table 2. HIV status was determined serologically. 

HPV-positivity among ESCC patients was not significantly associated with positive HIV status, when HPV-positive tumor patients were compared to HPV-negative tumor patients (0.0% vs. 12.9%; *p* = 1.0) and non-tumor patients (0.0% vs. 20.0%; *p* = 0.5)

HPV-positivity among ESCC patients was not significantly associated with smoking, when HPV-positive tumor patients were compared to HPV-negative tumor patients (80.0% vs. 46.4%; *p* = 0.3) and non-tumor patients (80.0% vs. 40.0%; *p* = 0.3). 

HPV-positivity in ESCC patients was not significantly associated with the consumption of alcohol, when HPV-positive tumor patients were compared to HPV-negative tumor patients (80.0% vs. 35.7%; *p* = 0.1) and non-tumor patients (80.0% vs. 20.0%; *p* = 0.09). There were significantly more patients who drank locally brewed alcohol among HPV-positive tumor patients compared to HPV-negative tumor patients (80.0% vs. 28.6%; *p* = 0.047) and non-tumor patients (80.0% vs. 10.0%; *p* = 0.02). 

## 3. Discussion

This study is, according to our knowledge, the first study combining PCR, in situ hybridization, Ki-67 index, and p16^INK4a^ immunohistochemistry, to investigate the role of HPV in ESCC carcinogenesis and the first study investigating HPV in ESCC in Malawi, a high-risk area for ESCC. 

The prevalence rate of HPV in both ESCC and benign esophageal papillomas, varies greatly all over the world [4,20] and even within the same high-risk area [21,22]. Such great geographic differences have not been reported for other HPV-associated malignancies, such as cervical cancer [9]. In our study, HPV prevalence was 15% in ESCC and HPV was found only in ESCC and dysplasia. No other HPV-type, except HPV16, was found. Similar to another local study from Zambia published in 2015, HPV was prevalent in a limited number of samples [23]. In Zambia, esophageal cancer is the fifth most common diagnosed cancer [1]. HPV was detected only in 2 out of 44 samples from ESCC patients using PCR [23]. A recent meta-analysis reported an overall prevalence of HPV in ESCC of 19.8% in Africa and 27.7% worldwide, with HPV16 being the most prevalent HPV type [20]. There was plausible variation across different detection methods and most of the studies used PCR for the detection of HPV [20,24]. PCR is known to be highly sensitive and, therefore, is not only susceptible to cross contamination, but also to substances inhibiting the reaction. This can lead to false-positive as well as false-negative HPV findings in tissue samples [16]. We have taken all possible precautionary measures to prevent the contamination of DNA during extraction and to ensure high quality DNA for PCR analyses. Despite over-fixation in buffered formalin, false-negative results are unlikely, as shown by *C*t-values ≤35 for amplified β-globin, which was used as an internal control. Reproducibility of the PCR results for HPV16 on different days was tested and showed high overall agreement (Fleiss’s Kappa = 0.6868, SE = 0.0348).

To define the prevalence of HPV within the histopathological context of the tumor samples and to determine viral load, quantitative PCR and HPV-DNA in situ hybridization was carried out. In cervical cancer, the HPV genome is usually present and transcriptionally active in every tumor cell, and a viral load of several hundred copies per cell has been reported [9,25]. Additionally, a distinct staining pattern, characteristic for the severity of neoplastic cervical lesions, is evident by in situ hybridization [26,27]. In our study, the average *C*t value for HPV16 in qPCR showed that HPV was not present in every cell and, if present at all, only a few viral genomes per cell persisted. Viral load exceeded 1.0 copy per cell in four of the tested HPV-positive samples. The maximum number of copies per cell was 2.5. This is consistent with results from another study examining viral load in ESCC with a multiplex real-time PCR assay. Si and colleagues found less than 10 copies per genome equivalent in 65% of the HPV16-positive esophageal cancer samples [28]. Reasons for the low viral load in ESCC samples may be the unequal distribution of HPV in the tumor tissue, or even loss of HPV during cancer development. Moreover, there is some evidence that low HPV copy numbers may be related to the physical state of the viral genome. Si and colleagues showed that the majority of ESCC samples harboured viral DNA in an integrated form [29]. The weakness of that study, however, was that the integration data was based on E2/E6 ratios, which provide only indirect evidence for HPV-integration. Thus far, sequencing of viral–cellular DNA junctions, and validation by PCR, has not been reported for ESCC. Integration of the viral genome may promote cellular growth and increase viral genome expression, compared to the extrachromosomal status of the viral genome [30]. 

In situ hybridization was carried out on 13 samples. In situ hybridization staining patterns among HPV-positive tumor samples did not differ from HPV-negative tumor samples (Table 1). This is in line with the determined low HPV-genome copy number per cell, which is below the detection limit of most in situ hybridization assays and excludes the presence of interspersed single cells with high copy numbers. Possibly, storage of the tissue in formaldehyde for several weeks before embedding in paraffin may have lowered sensitivity and worsened the background. This assumption seems likely since the control tissues, comprising cervical pre-cancer and cancer fixed in formaldehyde overnight, showed prominent and distinct hybridization signals (Figure S1, supplementary materials). Malik and colleagues tested p16-positive ESCC samples with in situ hybridization. None of the p16^INK4a^-positive samples showed high-risk HPV by in situ hybridization [31]. In another study, less than 10% of the cells showed positive weak staining in samples that tested positive by in situ hybridization [32]. Cooper and colleagues found HPV in 52% of ESCC samples by in situ hybridization. The virus was detected in isolated cells only, or aggregates of tumor cells, and the signals were punctuated in all HPV-positive tumors [33]. A punctuated signal pattern is reported for HPV-positive tumor cells with an integrated HPV genome [34]. 

To assess viral E7 oncogene activity in tumor samples, we performed p16^INK4a^ immunohistochemistry. Transforming viral infections are characterized by an upregulation of p16^INK4a^, due to the inactivation of Rb as a result of deregulated E7 gene expression. Strong, homogeneous and diffuse staining of p16^INK4a^ throughout the whole cancer tissue is a well-defined surrogate marker for HPV-induced cervical cancer [35]. In the literature, there is consent that, in contrast to cervical cancer, diffuse nuclear and/or cytoplasmic p16^INK4a^ staining of the entire lesion is not characteristic for ESCC. Unfortunately, the definition of p16^INK4a^-positivity in ESCC varies greatly and, thus, complicates direct comparison between studies. In our study, we showed that diffuse p16^INK4a^ staining of ≥20% of tumor cell areas was significantly associated with HPV positivity (*p* = 0.03). This correlation remained significant when raising the threshold level for p16^INK4a^-positivity to ≥50% of tumor cell areas (*p* = 0.03). Our results are consistent with another study investigating p16^INK4a^ expression in ESCC. Cao and colleagues found a correlation between HPV and p16^INK4a^ (86.2% p16^INK4a^-positive samples among HPV-positive tumors). Strong nuclear and/or cytoplasmatic staining over 70% was defined as positive in that study [36]. p16^INK4a^ staining of only parts of the tumor tissue is also described for tumors of other sites, such as vulvar neoplasias [37], carcinomas of the head and neck [38], anal cancers [39], and colorectal carcinomas [40]. Moreover, expression of p16^INK4a^ in tumor cells can be lost in the cells due to molecular events, such as promotor methylation or loss of heterozygosity [41]. 

However, there are several studies in which no correlation between p16^INK4a^-positivity and HPV status in ESCC was evident [15,31,32,42,43]. In those studies, the definition of p16^INK4a^-positivity ranged from ≥10% positive cells to 100%. Clearly, focal staining for p16^INK4a^, or even patchy staining, most likely reflects numerous physiological processes unrelated to HPV E7 expression [44]. However, it is of particular note that, in our study, the Ki-67 proliferation index in areas with diffuse nuclear or cytoplasmic p16^INK4a^ staining in at least 20% of tumor cell areas, or even in over 50% of tumor cell areas, was significantly higher in HPV-positive tumors than in the corresponding p16^INK4a^ stained areas of HPV-negative tumors (*p* = 0.003 and *p* = 0.004, respectively). It may be speculated that this increase in the Ki-67 proliferation index is due to the functional inactivation of Rb by the HPV E7 oncoprotein, which would provide support for a role of HPV in ESCC. No other published studies have compared the Ki-67 proliferation index in p16^INK4a^-positive tumor cell areas in relation to HPV status. Clearly, elevated levels of p16^INK4a^ expression are not exclusive for HPV-induced cancer. As reviewed by Witkiewicz and colleagues, there are two complementary models for the occurrence of tumors with high expression of p16^INK4a^. First, oncogenic stresses induce p16^INK4a^, thereby limiting tumorigenic progression. This is followed by the inactivation of Rb, which facilitates disease progression. Second, loss of Rb yields oncogenic stress, which induces p16^INK4a^ [45]. Since the Ki-67 proliferation index in p16^INK4a^ stained areas differed significantly between HPV-positive and HPV-negative tumors, the first model most likely applies for HPV-negative ESCC. This would align with the observations of Bai and colleagues, who showed that expression of p16^INK4a^ increases during esophageal squamous cell cancer progression [46]. 

The risk of developing cervical intraepithelial neoplasias increases in patients with an impaired immune system [9]. However, we found no association between HPV infection and HIV infection in patients with ESCC in Malawi. Significantly more HPV-positive tumor patients consumed locally brewed alcohol than non-tumor patients (*p* = 0.02) and HPV-negative tumor patients (*p* = 0.047). Locally brewed alcohol may act synergistically with the HPV infection in the pathogenesis of ESCC. The carcinogenic effect of locally brewed alcohol products could be mediated by acetaldehyde, the main metabolite of ethanol [47], but also by contaminants, such as fumonisins [48]. 

Limitations of our study include the low number of patients, especially the low number of HPV-positive tumor patients in the statistical analysis of further risk factors, and the long storage of the biopsies in formalin before embedding in paraffin. A self-generated questionnaire was used for the assessment of further risk factors, as no validated questionnaire for these factors was available in 2010. Not all questions could be answered by all patients. Furthermore, in Africa, patients with ESCC usually present themselves at a late stage of disease [49]. Therefore, only a few patients with early stages and dysplasias were included in this study. 

Several observations in our study, such as the presence of HPV16 DNA in cancers and dysplasias only, and the increased proliferation rate in p16^INK4a^-overexpressed tumor areas in HPV-positive cancers, suggest that HPV infection might play a role in a subset of ESCC patients. However, since the HPV genome does not seem to be present in every tumor cell, the theory of “hit and run” [50], in analogy to some β-HPV types, should also be considered for HPV-associated carcinogenesis in ESCC. This theory is discussed for HPV-initiated cutaneous squamous cell carcinomas and bovine papillomavirus type 4-initiated ESCC in cattle [51]. β-HPVs are found in epidermodysplasia verruciformis-associated cutaneous squamous cell carcinomas at UV-exposed skin sites. Furthermore, there is evidence that β-HPVs can act as co-factors in the development of UV-induced keratinocyte carcinomas in immunosuppressed individuals. A synergism between β-HPVs and DNA damage from UV light is suggested. β-HPVs in keratinocyte carcinoma are suggested to play a role during the early stages of carcinogenesis, by promoting the accumulation of UV-induced mutations and oncogenic transformation. Their persistence is not mandatory for the maintenance of the malignant phenotype and tumor viruses may be lost after initiation [51,52,53,54]. Studies investigating human skin biopsies have shown low viral loads in squamous cell carcinomas examined by qPCR assays and confirmed by in situ hybridization, showing only few positive nuclei per section [55]. In β-HPV-associated skin lesions, HPV status and p16^INK4a^ expression do not correlate [56]. In cervical cancer, the continued viral oncogene activity of high risk HPV types of the α genus is characteristic. However, this mechanism may not be exclusive, in particular, when considering the carcinogenic role of HPV16 in other epithelial sites such as the oesophagus. Indeed, 47.4% of ESCC display somatic TP53 mutations [57], which would be in line with a “hit and run” mechanism.

In conclusion, the role of human papillomaviruses in the carcinogenesis of esophageal squamous cell carcinomas, still remains elusive. To further investigate the role of HPV in ESCC, more studies, with a stronger focus on molecular events in the context of intratumoral heterogeneity and other risk factors, need to be conducted.

## 4. Material and Methods

Fifty-five patients receiving diagnostic upper gastrointestinal endoscopy at Zomba Central Hospital or Queen Elizabeth Hospital in Blantyre in 2010, were included in our study. Biopsies of all 55 patients were taken and immediately fixed in buffered formalin. Sera were collected from 42 patients. Further investigation was done at Jena University Hospital, where the biopsies were embedded in paraffin. Formalin-fixed paraffin-embedded (FFPE) tissue blocks were cut as consecutive sections. The first and the last sections (3 µm) were stained with hematoxylin and eosin for histopathological diagnosis. The sections in-between (15 µm) were used for DNA extraction and subsequent qPCR. Renewed sectioning was performed for in situ hybridization and immunostaining for p16^INK4a^ and Ki-67. 

All patients gave their written consent prior to endoscopy (signature or finger print). The study was approved by the College of Medicine Research and Ethics Committee (COMREC) (approved 21 May 2010, Nr P.04/10/930). 

### 4.1. DNA Extraction

Consecutive serial tissue sections were prepared for DNA extraction. The first and last sections were hematoxylin-eosin-stained for histopathological diagnosis and to estimate the fraction of tumor cells in the tissue. Ten 15 µm tissue sections were deparaffinized with xylene, re-hydrated through a graded ethanol series, and dissolved in a 500 µL digestion buffer (100 mM NaCl, 10 mM Tris-HCl pH8.0, 25 mM EDTA (Ethylenediaminetetraacetic acid), and 0.5% SDS (Sodium dodecyl sulfate)). Proteinase K (100 µg/mL) digestion was done overnight at 56 °C. For DNA extraction, an equal volume of phenol/chloroform/isoamylalcohol was added and vortexed intermittently for 30 s. After centrifugation, the DNA in the aqueous phase was precipitated by adding 1/10th volume of 7.5 M ammonium acetate and 2 volumes of ice-cold ethanol, followed by another centrifugation step. The pellet was dissolved in 25 µL 10 mM Tris/HCl pH = 8.0. DNA concentration and quality were determined by spectrophotometry (NanoDrop ND-1000, Thermo Fisher Scientific GmbH, Dreieich, Germany).

### 4.2. Multiplex Real-Time PCR Assay (TaqMan Format)

A multiplex real-time PCR assay (TaqMan format [58]), designed to detect and quantify the 7 most frequent high-risk HPV types (HPV16, 18, 31, 33, 45, 52, and 58) in cervical cancer, was used. It was conducted as described previously [59]. Reactions were run in an ABI 7300 cycler (Applied Biosystems, Darmstadt, Germany), which is able to detect 4 different fluorescent dyes simultaneously. Reaction mix 1 detects HPV16, 18, 31, and 45, and reaction mix 2 detects HPV33, 52, 58, and β-globin. The PCR primers targeted the LCR/E6/E7 regions of the HPV genomes and the PCR products were detected by the corresponding TaqMan probes. DNA quality and the relative viral copy number were determined by amplifying the housekeeping gene *β-globin* in one of the multiplex reactions. PCR was performed in an end volume of 25 µL, containing 9.5 µL DNA (total of 50 ng), 12.5 µL Platinum Quantitative PCR SuperMix-UDG (Invitrogen, Carlsbad, CA, USA) to prevent amplification of carry-over products [60], 10 pmol of each primer, and 1–5 pmol of each probe. Forty-five PCR cycles were run at 94 °C for 15 s, 50 °C for 20 s, and 60 °C for 40 s, each [59].

Reproducibility of the PCR results was assessed by repeating the assay on different days. All samples were tested for HPV16-positivity at least three times. All samples were tested with a primer mixture of reaction 1 twice, and once with a primer mixture containing primers for HPV16 and β-globin only (duplex PCR). For 30 samples, there was enough DNA left to repeat the duplex PCR assay twice (Table 3). 

*C*t values of the HPV16 genome and β-globin were used to calculate the viral load of HPV16, as copies per cell in 6 of 7 HPV-positive samples, according to the following formula:

$$\left(\frac{1}{2^{\Delta Ct}}\right)÷2$$

To assess validity, viral load was determined the same way in HPV16-positive cell lines with a known viral load: SiHa (*C*t HPV16: 21.8, *C*t β-globin: 23.93, viral load: 2.2 copies per cell), HPKII (*C*t HPV16: 21.48, *C*t β-globin: 24.77, viral load: 5.0 copies per cell), and MRI (*C*t HPV16: 19.07, *C*t β-globin: 24.96, viral load: 29.7 copies per cell).

Fifteen samples were of sufficient size for renewed sectioning and DNA extraction. These samples were analysed for all 7 hrHPV types (Table 3).

### 4.3. In Situ Hybridization 

The FFPE sections were deparaffinized with xylene, re-hydrated through a graded ethanol series, and washed in phosphate buffered saline (PBS). In situ hybridization was conducted using Dako GenPoint HPV Biotinylated DNA probe according to the manufacturer’s protocol (GenPoint/Dako Cytomation, Agilent, Waldbronn, Germany). GenPoint HPV Probe contains HPV genomic clones in the form of double-stranded fragments of 500 base pairs or less and multiple biotinylated oligonucleotides from 25 to 40 bases in length. Briefly, the deparaffinized tissue sections were incubated with 0.8% pepsin at 37 °C for 5–10 min, rinsed in deionized water, immersed in 0.3% H_2_O_2_ in methanol for 20 min, and rinsed in deionized water again. A drop of GenPoint HPV Biotinylated DNA probe was added to the air-dried sections. After heat denaturation at 90 °C, hybridization was done in a pre-warmed humid chamber at 37 °C for 16 to 18 h. The slides were washed in Tris-buffered saline with Tween20 (TBST). Hybridized probes were detected by streptavidin-HRP. 

### 4.4. p16^INK4a^

The expression of p16 was analysed by immunohistochemistry. A CINtec Histology Kit (biogen/Roche, Tucson, AZ, USA) containing the monoclonal antibody anti-p16^INK4a^ (E6H4) was used according to the manufacturer’s protocol for qualitative detection of p16^INK4a^ proteins in FFPE tissue sections. After adding the substrate-chromogen solution, slides were washed with distilled water and stained with Harris Hematoxylin, followed by washing with tap water. Finally, the sections were dehydrated and mounted with coverslips. Similar to cervical precancers (CIN2/3) and cancer, samples showing a diffuse nuclear p16^INK4a^ staining pattern were considered to be positive. Furthermore, samples showing a diffuse cytoplasmic p16^INK4a^ staining pattern were also defined as p16^INK4a^-positive. However, in contrast to cervical cancer, p16^INK4a^ staining showed considerable intratumoral heterogeneity. To account for this, p16^INK4a^-positivity was scored in the context of all tumor cell areas, i.e., biopsies in which 20–50% and ˃50% of tumor cell areas showed diffuse staining were scored as positive and strongly positive, respectively. Biopsies with focal staining or with less than 20% diffuse staining were defined as p16^INK4a^-negative. 

### 4.5. Ki-67

To assess the proliferation activity of tumor cells, the Ki-67 index was determined. Ki-67-positive cells were counted in the area with the highest Ki-67 staining. In addition to this overall Ki-67 index, we determined the Ki-67 index in p16^INK4a^-positive areas. At least 200–300 cells were counted. Ki-67 staining indices were grouped into negative (<10%), weakly positive (10–20%), positive (20–50%), and strongly positive (>50%).

### 4.6. Serological Analysis (ELISA)

The HIV status of each patient was determined using a commercially available ELISA system (Enzygnost^®^ HIV Integral II, Siemens, Erlangen, Germany) according to the manufacturer’s protocol. The tests were performed in a BEPIII system (Siemens, Germany). Both HIV specific antibodies and the HIV p24 antigen, were detected, ensuring very high diagnostic sensitivity. 

### 4.7. Data on Alcohol Consumption and Smoking, and Clinical Data 

Data on the consumption of alcohol and tobacco, as well as clinical data such as history of Herpes zoster, tuberculosis (TBC), and oral thrush, were collected using a self-generated questionnaire. Not all questions could be answered by all patients. 

### 4.8. Pathological Analysis

Histopathological diagnosis, grading and analysis of p16^INK4a^, and Ki-67 immunohistochemistry were done by ALG and an experienced pathologist (AG) at Jena University Hospital. 

### 4.9. Statistical Analysis

To assess the relationship between the epidemiological data and clinical data, an exact Fisher test was used for nominal data and a Mann–Whitney U test was used for metric data. Therefore, median and interquartile ranges were determined to describe the variance of the metric variables. Results were considered statistically significant when *p* < 0.05. To test the reproducibility of the PCR results, Fleiss’s Kappa was assessed. Statistical analysis was performed with IBM SPSS Statistics 22. 

## Figures and Tables

**Table 1:** Human papillomavirus (HPV), human immunodeficiency virus (HIV), p16, and Ki-67 status in esophageal squamous cell carcinoma (ESCC), dysplasia and histopathologically normal biopsies.

Study Number	Hist. Diagn. ^1^	Grading	Ki-67 Index Overall ^2^	p16^INK4a^ Staining Pattern ^3^	p16^INK4a^ Stained Tumor Cell Areas ^4^	% of Ki-67 Stained Cells within p16^INK4a^ Stained Areas	HPV qPCR ^5^	HPV ISH ^6^	HIV Status ^7^
HV090610-44	1	1	3	2	1	71	1	0	0
DV280610-68	1	1	3	1	2	88	1	0	0
EC070710-73	2	-	3	1	2	51	1	0	0
FN280710-105	1	1	3	1	2	67	1	0	-
AM240510-22	1	2	3	1	2	61	1	0	0
JS070710-72	1	1	3	1	2	72	1	0	0
MRD160610-57	1	2	-	-	-	-	1	-	0
PD120510-1	1	2	3	0	0	-	0	0	0
CC050710-70	2	-	3	0	0	-	0	0	1
AM14071087	1	2	2	2	2	26	0	0	0
EK070610-40	1	2	3	0	0	-	0	-	0
SN210710-101	1	2	3	0	0	-	0	-	-
SN210710-96	1	2	3	0	0	-	0	-	-
HM070710-74	1	3	-	-	-	-	0	-	0
LI190710-91	1	2	-	-	-	-	0	-	-
DM090610-41	1	2	3	1	2	60	0	-	0
AOM160610-55	1	2	3	1	2	53	0	0	0
MG260510-29	1	2	3	1	2	55	0	-	0
EO160610-59	1	3	3	1	1	60	0	-	0
LC150610-97	1	2	2	1	2	25	0	-	-
LM180510-10	1	2	3	0	0	-	0	-	1
PL130710-81	1	2	2	1	2	46	0	0	0
RC170510-7	1	2	2	1	2	45	0	-	0
EM280610-69	2	-	3	1	2	67	0	-	0
LN180510-12	1	3	2	0	0	-	0	-	-
ESL190510-17	1	3	2	1	2	48	0	-	0
JC260710-102	1	2	3	0	0	-	0	-	-
DL160610-56	1	3	2	1	2	49	0	-	1
JC120510-2	1	2	3	0	0	-	0	-	0
JN140610-54	1	2	0	1	1	0	0	0	0
RM170510-8	1	2	2	1	2	47	0	-	0
ZV160610-58	1	2	3	1	1	62	0	-	0
SF270710-103	1	-	2	0	0	-	0	-	-
FM140710-86	1	3	3	1	2	54	0	-	0
OD120510-3	1	2	2	1	2	45	0	-	0
DK280610-66	1	3	3	1	1	78	0	-	0
MJ020610-34	1	3	2	0	0	-	0	-	1
JK140710-89	1	2	3	1	2	65	0	-	0
RM210710-93	1	2	3	0	0	-	0	-	-
AM140710-84	1	3	3	0	0	-	0	-	0
BZ020610-35	1	2	0	1	2	8	0	0	0
GP140710-90	1	2	2	1	1	41	0	-	-
JC070710-77	1	2	3	2	2	28	0	-	0
RM150610-98	0	-	2	0	0	-	0	-	0
MJ240510-23	0	-	3	0	0	-	0	-	1
EC070710-73	0	-	2	0	0	-	0	-	-
DD170510-9	0	-	3	0	0	-	0	-	0
FN280610-62	0	-	2	0	0	-	0	-	0
AM140610-50	0	-	2	0	0	-	0	-	1
KG170510-4	0	-	1	0	0	-	0	-	1
AD130710-83	0	-	2	0	0	-	0	-	0
AT090610-45	0	-	1	0	0	-	0	-	0
GN280710-107	0	-	2	0	0	-	0	-	-
MB140610-51	0	-	3	0	0	-	0	-	0
JM130710-82	0	-	0	1	1	6	0	-	-

^1^ Histological diagnosis: 0 = tumor-free epithelium, 1 = ESCC, 2 = dysplastic epithelium; ^2^ Ki-67 index: 0 ≤ 10%: negative, 1 = 10–20%: weakly positive, 2 = 20–50%: positive, 3 ≥ 50%: strongly positive; ^3^ p16^INK4a^ staining pattern: 0 = focal staining, 1 = diffuse cytoplasmatic positive, 2 = diffuse nuclear positive; ^4^ p16^INK4a^ staining in tumor tissue: 0 = p16^INK4a^ negative, 1 = 20–50% positive, 2 ≥ 50% strong positive; ^5^ HPV in PCR: 0 = negative, 1 = positive; ^6^ In situ hybridization: 0 = negative; ^7^ HIV status: 0 = negative, 1 = positive; - not done/not evaluable.

**Table 2:** Clinico-pathological characteristics of patients.

Patients’ Characteristics	HPV-Positive Tumor Patients (*n* = 6)	HPV-Negative Tumor Patients (*n* = 34)	*P* Value *	Non-Tumor Patients (*n* = 12)	*P* Value **
**General**					
Age (years)	50.0 (13.0)	50.5 (28.0)	0.7	43.5 (38.0)	0.7
Male gender	5 (83.3%)	22 (64.7%)	0.6	9 (81.8%)	1.0
**Risk factors**					
Oral thrush in history	1 (20.0%)	4 (14.3%)	1.0	1 (10.0%)	1.0
Tuberculosis in history	0 (0.0%)	4 (14.3%)	1.0	0 (0.0%)	a
Herpes zoster in history	0 (0.0%)	2 (7.1%)	1.0	0 (0.0%)	a
HIV-positive	0 (0.0%)	4 (12.9%)	1.0	2 (20.0%)	0.5
Smoker	4 (80.0%)	13 (46.4%)	0.3	4 (40.0%)	0.3
Pack years	3.0 (12.0)	0.0 (4.0)	0.1	0.0 (1.0)	0.05
Duration of smoking (years)	15.0 (30.0)	0.0 (9.0)	0.08	0.0 (4.0)	0.04
Number of cigarettes	6.0 (7.0)	0.0 (6.0)	0.2	0.0 (5.0)	0.05
Alcohol	4 (80.0%)	10 (35.7%)	0.1	2 (20.0%)	0.09
Locally brewed alcohol	4 (80.0%)	8 (28.6%)	0.047	1 (10.0%)	0.02
Duration of drinking alcohol (years)	17.0 (21.0)	0.0 (5.0)	0.1	0.0 (1.0)	0.02

Data are presented as absolute numbers with percentages in brackets or medians with interquartile ranges in brackets. * Comparison of HPV-positive tumor patients with HPV-negative tumor patients. ** Comparison of HPV-positive tumor patients with non-tumor patients. (a) An exact Fisher test was not performed as the variable is a constant.

**Table 3:** HPV DNA detection by multiplex PCR.

PCR Primer Mixture		Run	Number of Tested Samples
Mixture 1	HPV16, 18, 31, 45	1	55
		2	55
Duplex only	HPV16 and β-globin	1	55
		2	30
		3	30
Mixture 1 and 2	HPV16, 18, 31, 45 and HPV33, 52, 58 and β-globin	1	15

## Supplementary Materials

The following are available online at http://www.mdpi.com/1422-0067/19/2/557/s1.

## Abbreviations

HPV	Human papillomavirus
ESCC	Esophageal squamous cell carcinoma
Rb	Retinoblastoma
qPCR	Quantitative PCR
TBC	Tuberculosis
HIV	Human immunodeficiency virus
