# Supplementary material for: Detection of HPV16 in Esophageal Cancer in a High-Incidence Region of Malawi
Source: Int J Mol Sci. 2018 Feb 12;19(2):557. doi: 10.3390/ijms19020557 (PMC5855779; doi:10.3390/ijms19020557)
Supplement: Supplementary file 1 [file ijms-19-00557-s001.pdf]

Supplementary material

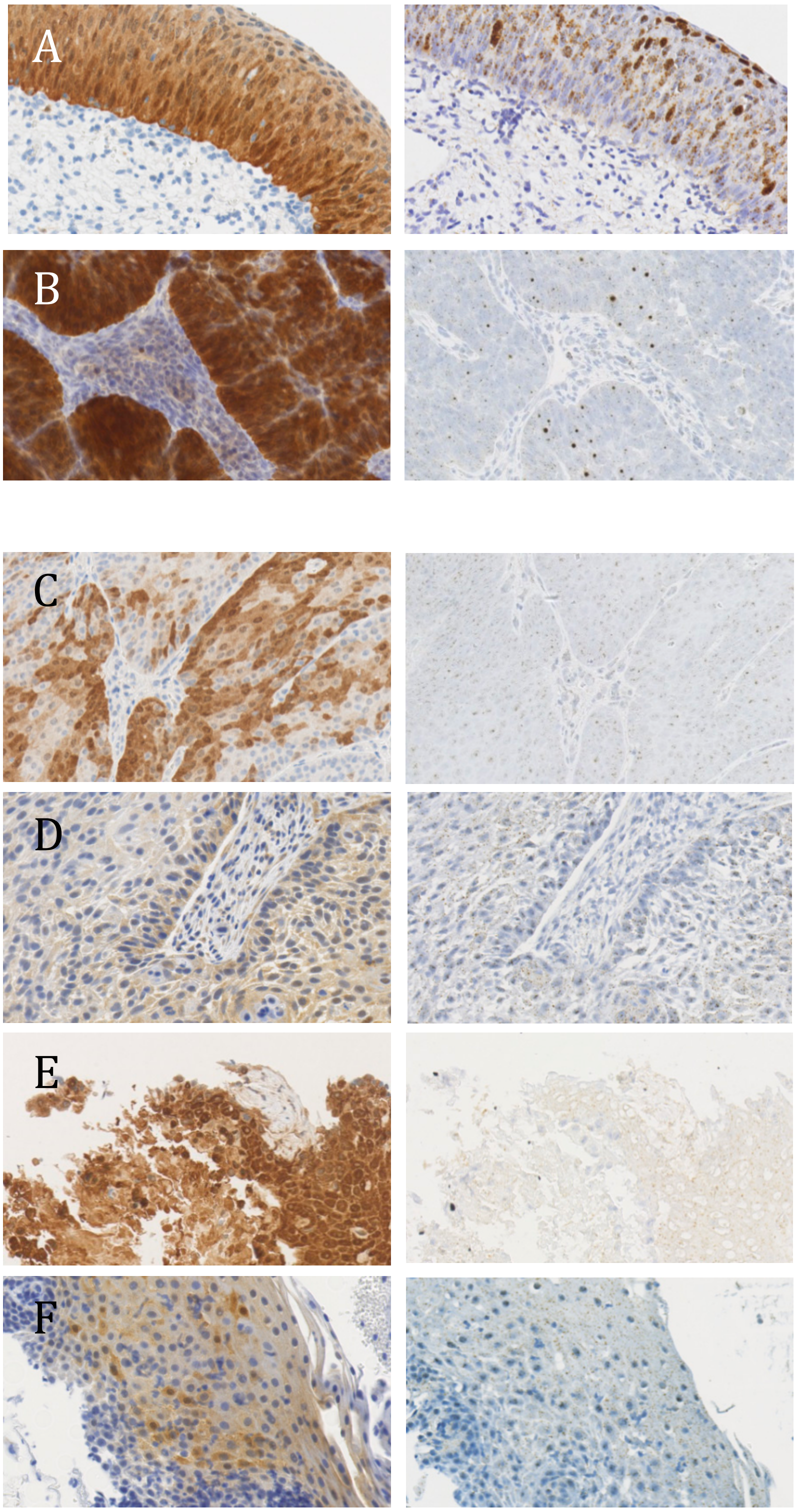

Figure S1: p16^{INK4a} staining and HPV16 in situ hybridization (left and right panel, respectively) in consecutive sections of control tissues (upper panel) and ESCC (lower panel). Distinct ISH signals are only evident for the control tissues. A: HPV positive high grade cervical intraepithelial lesion. B: HPV positive cervical carcinoma. C: HPV positive, diffuse nuclear p16^{INK4a} positive ESCC. D: HPV positive, diffuse cytoplasmic positive ESCC. E: HPV negative, diffuse nuclear p16^{INK4a} positive ESCC. F: HPV negative, diffuse cytoplasmatic p16^{INK4a} positive ESCC.
